# Novel models for prediction of benefit and toxicity with FOLFIRINOX treatment of pancreatic cancer using clinically available parameters

**DOI:** 10.1371/journal.pone.0206688

**Published:** 2018-11-09

**Authors:** Konstantin Schlick, Teresa Magnes, Lukas Ratzinger, Bernhard Jaud, Lukas Weiss, Thomas Melchardt, Richard Greil, Alexander Egle

**Affiliations:** 1 IIIrd Medical Department with Hematology and Medical Oncology, Hemostaseology, Rheumatology and Infectious Diseases, Oncologic Center, Paracelsus Medical University, Salzburg, Austria; 2 Salzburg Cancer Research Institute, Salzburg, Austria; 3 Cancer Cluster Salzburg, Salzburg, Austria; University of South Alabama Mitchell Cancer Institute, UNITED STATES

## Abstract

**Background:**

Despite modern chemotherapy regimens, survival of pancreatic cancer patients remains dismal. Toxicity is a major concern and it is a challenge to upfront identify patients with the highest benefit from aggressive polychemotherapy. We aimed to evaluate ORR and side effects of the FOLFIRINOX regimen, highlighting dose modification and to explore possible prognostic response factors as a clinical tool.

**Methods:**

This retrospective study includes 123 patients with metastatic PC that were treated with FOLFIRINOX between the years 2007 to 2016 in a single academic institution. Survival rates were analysed using the Kaplan-Meier method. Prognostic models including laboratory and clinical parameters were calculated using Cox proportional models in univariate and multivariate analyses.

**Results:**

Median age at diagnosis was 64 years (47–78 years), 71 (57, 7%) were male and the majority had an ECOG performance status of 0 or 1 (63 patients; 83.7%). After a median follow up of 17.8 months, median progression free survival (PFS) and overall survival (OS) were 5.7 (4.55–6.84; 95%CI) and 11.8 months (9.35–14.24; 95%CI) respectively. Overall response rate with FOLFIRINOX was 34.9% and stable disease rate was 21.9%. Regarding Grade 3/4 side effects, 62 events, were reported in 37 patients. Looking at risk factors e.g. patient characteristics, tumor marker, inflammatory markers and body composition multivariate analyses proved CEA >4 elevation and BMI > 25 at the time point before palliative chemotherapy to be independent negative prognostic factors for OS. Grouping patients with no risk factor, one or two of these risk factors we analyzed a median OS of 17.4 moths, 9.6 months and 6.7 months (p<0.001) respectively. In addition we identified thrombocytosis and low BMI as predictors of early toxicity.

**Conclusion:**

This study identifies two easily available factors influencing overall survival with FOLFIRINOX therapy. By combining these two factors to create a score for OS, we propose a prognostic tool for physicians to identify patients, who are unlikely to benefit more from FOLFIRINOX or likely to experience toxicity.

## Introduction

Pancreatic cancer (PC) remains one of among the most devastating malignant disease and outcome is dismal with a prognostic 5-year survival rate of only 5% [1]. In an analysis of the Cancer Treatment Centers of America 64% of patients diagnosed between 2004 and 2008 were alive at 6 months, 32% at 1 year and 17% at 1,5 years. OS rates were largely unchanged in an analysis of the same institution carried out between 2000 and 2011. PC is the fourth leading cause of cancer related death in the United States in 2017 even with the introduction of novel systemic chemotherapy options [1] and its contribution to cancer mortality is expected to surpass that of colorectal cancer in 2020, then ranking 2^nd^ after lung cancer [2]. A curative treatment approach with a radical surgical resection can only be offered to less than 20% of cases as most patients are in advanced stage of disease and the vast majority of patients present with incurable disease. [3]. Recently trials like ACCORD and MPACT, could show improved overall survival (OS) with the use of combined chemotherapy modalities. [4,5]. The MPACT trial demonstrated an overall survival benefit with gemcitabine and nab-paclitaxel over gemcitabine monotherapy of 8.7 months compared to 6.6 months respectively. The ACCORD trial reported a median overall survival of FOLFIRINOX of 11.1 months compared to 6.8 months with the standard of care gemcitabine monotherapy. Currently this is the largest survival advantage shown in any clinical phase III trial for PC. However severe side effects, Grade 3–4, quite frequently occur and raise questions of patient selection, asking for dose-reduced or dose-modified schedules [6]. Proper patient selection is crucial to identify those that are most likely to benefit from aggressive chemotherapy approaches and also separate them, who will likely have only little benefit due to increased rates of severe side effects. However, no prospectively validated models are available to guide decision making for an upfront patient identification. Therefore we conducted a retrospective review of patients with metastatic or unresectable pancreatic cancer treated with FOLFIRINOX in a daily practice of a single academic center and to develop a new prognostic risk model.

## Materials and methods

### Patients selection and data acquisition

We performed a retrospective analysis from patients with diagnosis of PC treated at the III^Ird^ Medical Department of the Paracelsus Medical University Salzburg. All patients beyond 18 years of age and treated with FOLFIRINOX between January 2007 and Mai 2016 for pathologically or imaging confirmed locally advanced or metastatic pancreatic adenocarcinoma were eligible for inclusion. Treatment with at least one cycle of 5-fluorouracil (5-Fu), oxaliplatin and irinotecan was required for inclusion into this analysis. No patients were still on treatment with FOLFIRINOX at the time point of data analysis.

The polychemotherapy FOLFIRINOX regimen was used in all patients as previously described in the ACCORD 11 trial [4] (Conroy NEJM 2011,May12.) and consisted of oxaliplatin 85mg/m, leucoverin 400mg/m, irinotecan at 180mg/m and fluorouracil at 400mg/m^2^ followed by continuous infusion of 2400mg/m^2^ over 46h by a period of 2 weeks. One therapy cycle contains 2 dose applications on days 1 and 15.

Dose modifications were made at the discretion of the treating physician. As per clinical standard physicians include performance status and comorbidities into primary dose modification decisions and mostly toxicity events in secondary dose modifications. As per our institutional standard treatment was continued until disease progression or documentation of unacceptable toxicities. We retrospectively evaluated patient characteristics, ECOG performance score, date of diagnosis, start of FOLFIRINOX treatment, dose modifications during treatment, toxicity, response rates, progression free survival (PFS) and overall survival (OS) based on review of patient’s medical records and radiology reports. ECOG score was measured according to the World Health Organization [7]. Further second line or additional therapy was extracted if applicable.(see S1 Table) Patients were evaluated for toxicities at the start of each cycle with history, examination, performance status, complete blood count and serum markers.

Patients routinely received 5-HT3 antagonists and dexamethasone as a standard of care for emesis prophylaxis.

The four most common adverse events of FOLFIRINOX, reported in the literature, are leucopenia, diarrhea, polyneuropathy (PNP) and infectious complications.

For our analyses we concentrated on documenting only the clinical consequential side effects, respectively G 3/4 toxicity, of these four adverse events mentioned above, by assessing chart review, which were graduated according to National Cancer Institute Common Terminology Criteria for Adverse Events (CTCAE) [8].

Tumor response was defined using CT scans classified into partial response, stable disease or progressive disease according to WHO criteria and by tumor marker response [9].

PFS was defined as the time from start of FOLFIRINOX until date of progress or death from any cause and OS was as defined from start of FOLFIRINOX until death from any cause.

BMI groups were defined according to the WHO guidelines according to height and weight at start of chemotherapy: underweight (BMI<18.5kg/m2), normal weight (BMI 18.5 to<25kg/m2), overweight (BMI 25 to >30kg/m2) and obesity (BMI>30kg/m2) [10].

### Statistics

Data analysis for this retrospective study was descriptive in nature and presented in means, medians and ranges (95% CI). PFS and OS estimates were obtained using the Kaplan-Meier (KM) method. The median follow-up duration was measured by reverse KM estimator. For association of OS we initially performed a univariate assessment of predefined prognostic parameters (See Table 1) comparing survival curves by the use of Cox regression univariate analyses including hazard ratio (HR) with 95% confidence interval (CI). Multivariate Cox regression analyses for OS was then calculated using all significant variables from the univariate analyses. The optimal risk factor parameters cut-off values were also calculated based on the receiver operating characteristics (ROC) analyses and the Yoden Index J, which represents the maximum sensitivity and specificity for all cut points in the ROC curve [11]. All statistical analyses were performed with a statistical software package (SPSS, version 21 IBM Corp). A p-value of < 0.05 was considered to be statistically significant in regard to median OS with 95% CI.

#### Ethics statement

This analysis was approved the Ethics Committee of the provincial of Salzburg Austria and waived the requirement for informed consent. All patients records were fully anonymized. At the time point of data analyzation no patient was alive any more.

## Results

### Patients

Between January 2008 and May 2016 a total number of 375 patients with the diagnosis of pancreatic cancer were seen at our department. Within this group of patients, 123 received FOLFIRINOX, 35.8% (n = 44) for LAPC and 64.2% (n = 79) for MPC. Regarding primary site of metastatic disease of the 84 patients, 43 patient had liver metastases only compared to 11 patients with extra hepatic metastasis and 26 patients with both hepatic and extrahepatic metastasis. There was no statistically significant difference in OS regarding these three groups (p = 0.105).

Patients were followed for a median of 17.85 months (range: 0.43–55.23 months). A treatment approach in curative intent with resection, at the time of initial diagnosis, was performed in n = 22 (17.9%) of the patients and in 18 (14.6%) of the patients adjuvant gemcitabine as the state of the art adjuvant therapy at that time was offered. Furthermore, a neo-adjuvant therapy setting including FOLFIRINOX was applied in 4 patients (3.3%) before receiving the FOLFRIRNOX regimen in the palliative therapy setting later on. All other 79 (64.2%) received FOLFIRINOX in a primary palliative treatment setting.

All 123 patients were treated with at least 1 dose of FOLFIRINOX and were eligible for our analyses. Patient’s characteristics are shown in detail in Table 1. The median age at diagnosis was 64 years (47–78 years), 71 (57, 7%) were male and the majority had an ECOG performance status of 0 or 1 (n = 63; 83.7%) as expected for a selection for polychemotherapy.

**Table 1:** Characteristics of the 123 patients treated with FOLFIRINOX.

Characteristic	Score(N = 123)
**Age–no. of yr**	
**Median**	64
**Range**	47–78
**Sex–no. (%)r**	
**Male**	71 (58%)
**Female**	52 (42%)
ECOG* performance Status–no. (%)	
**0 or 1**	63 (51.2%)
**2 or 3**	60 (48.8%)
**Biliary stent–no. (%)**	
**yes**	17 (13.5%)
**no**	106 (86.5%)
**Carbohydrat antigen 19–9 elevation (>35 U/ml)–no. (%)**	
**yes**	91 (73.8%)
**no**	32 (26.2%)
**Carcinoembryonic antigen elevation (>4 mcg/l)–no. (%)**	
**yes**	92 (75%)
**no**	31 (25%)
**Tumor stage at Diagnosis–no (%)**	
**Locally advanced**	44 (36%)
**Metastatic**	79 (64%)
**-hepatic**	42 (52%)
**-extra hepatic**	11 (13%)
**-both**	26 (35%)
**Objektive Response–no (%)**	
**Partial response (PR)**	43 (35%)
**Stable disease (SD)**	27 (22%)
**Progressive disease (PD)**	38 (31%)
**No follow up**	15 (12%)
**Second line chemotherapy**	
**Gemcitabine**	12 (10%)
**Gemcitabine/Nab-Paclitaxel**	43 (34.9%)
**Other**	33 (26.8)
**No second line chemotherapy**	35 (29%)

* Eastern Cooperative Oncology Group

### Treatment

A total of 1,093 doses were administered in 123 patients (100%) treated with FOLFIRINOX with a median number of doses of seven (range: 1–36) and of 3.5 cycles per patient. Ten patients (12.3%) received only one dose and 38 (30.8%) of the patients received less than 4 applications either due to disease progression or poor tolerance. 30% of the patients (n = 37) received more than 12 applications. Chemotherapy dose modification was left at the discretion of the treating physician according to comorbidities, expected and observed toxicities.

Only 81 (66%) of the patients started with full dose of all drugs included in the polychemotherapy regiment FOLFIRINOX. 45/ 81 (55.3%) received full doses throughout their whole treatment period without severe adverse events necessitating dose reduction or cessation.

In contrast, in 42 (34%) of the patients treatment was started with a modified first application defined by dose reduction of at least one substance up to a maximum of 25%.

In the full dosage-starting cohort we detected 40 Grade 3–4 events of the specified types compared to 22 events in the dose-modified group (p<0.01).

Dose delays greater than seven days occurred in 85 (69.1%) of all patients patients, with 26 (21%) having two and 22 (17.8%) of all patients (n = 22) requiring three or more doses delayed.

### Clinical outcome

Tumor response was assessed by imaging tests according to WHO criteria as well as CEA and CA 19–9 tumor marker response. Tumor markers were routinely assessed at baseline and before each treatment cycle. Disease control, including partial remission and stable disease, was achieved in 70 (56.9%). At three months 30.8% of the patients (n = 38) had primary progressive disease and 12% of the patients (n = 15) could not be evaluated due to missing imaging for response. After a median follow up of 17.8 months the median PFS and OS for the whole cohort was 5.7 months and 11.8 months respectively. Patients with LAPC showed a PFS of 5.6 months and an OS of 11.8 months, and patients with MPC had a median of 3.3 months for PFS and of 4.4 months for OS. However, differences between LPAC and MPAC were not statistically significant.

### Subsequent treatment after FOLFIRINOX

In order to maintain remission status, 17 (20.9%) patients received oral capecitabine or local radiotherapy 6.5% (n = 8) after cessation of FOLFIRINOX.

In progression after FOLFIRINOX a 2^nd^ line chemotherapy was initiated in 88 (71%) of the patients. The most widely used regimen was gemcitabine-Nabpaclitaxel in 34.9% of the patients (n = 43), 4.8% of the patients (n = 6) received gemcitabine in combination with erlotinib as a doublet, 10.5% of the patients (n = 13) were treated with gemcitabine monotherapy and 21.1% of the patients (n = 26) received therapy with other cytotoxic agents (see complete list of applied chemotherapeutic agents in S1 Table).

A third line palliative chemotherapy was offered to 32% of the patients (n = 40) with 27% of the patients (n = 22 patients) receiving either docetaxel or doxorubicin respectively.

Seven patients underwent surgery in palliative intention in order to get control over symptomatic hepatic metastatic or for local tumor debulking.

### Prognostic factor analyses

To identify prognostic factors for patients, who may have only minimal benefit of FOLFIRINOX treatment we analyzed the following parameters at the time of diagnosis with respect to predicting outcome: age and ECOG at diagnosis, gender, stage of disease, CEA, CA19-9, bilirubin, CRP, leukocytes, neutrophils, lymphocytes, monocytes, platelets, hemoglobin, and BMI. An elevated tumor marker was present in 75% (n = 61) and 73.8% of the patients (n = 60) for CEA and Ca19-9, respectively. A further 55.3% of the patients (n = 45) had an elevated CRP detected and 30.75% of the patients (n = 25) had a BMI >25. BMI distribution through the cohort was underweight 8.9% of the patients (n = 11), normal weight 70.7% of the patients (n = 87) and overweight 30.75% of the patients (n = 25)

In univariate analyses we found that CEA>4 (p <0.001), Ca19.9 >400 (p = 0.049), CRP (p = 0.049) and BMI>25 (p = 0.002) were significantly associated with OS.

Thus, a multivariate regression analysis was conducted including these four parameters and we could confirm an independent prognostic value for CEA > 4 (p = 0.013) and BMI >25 (p = 0.017).

**Fig 1:**
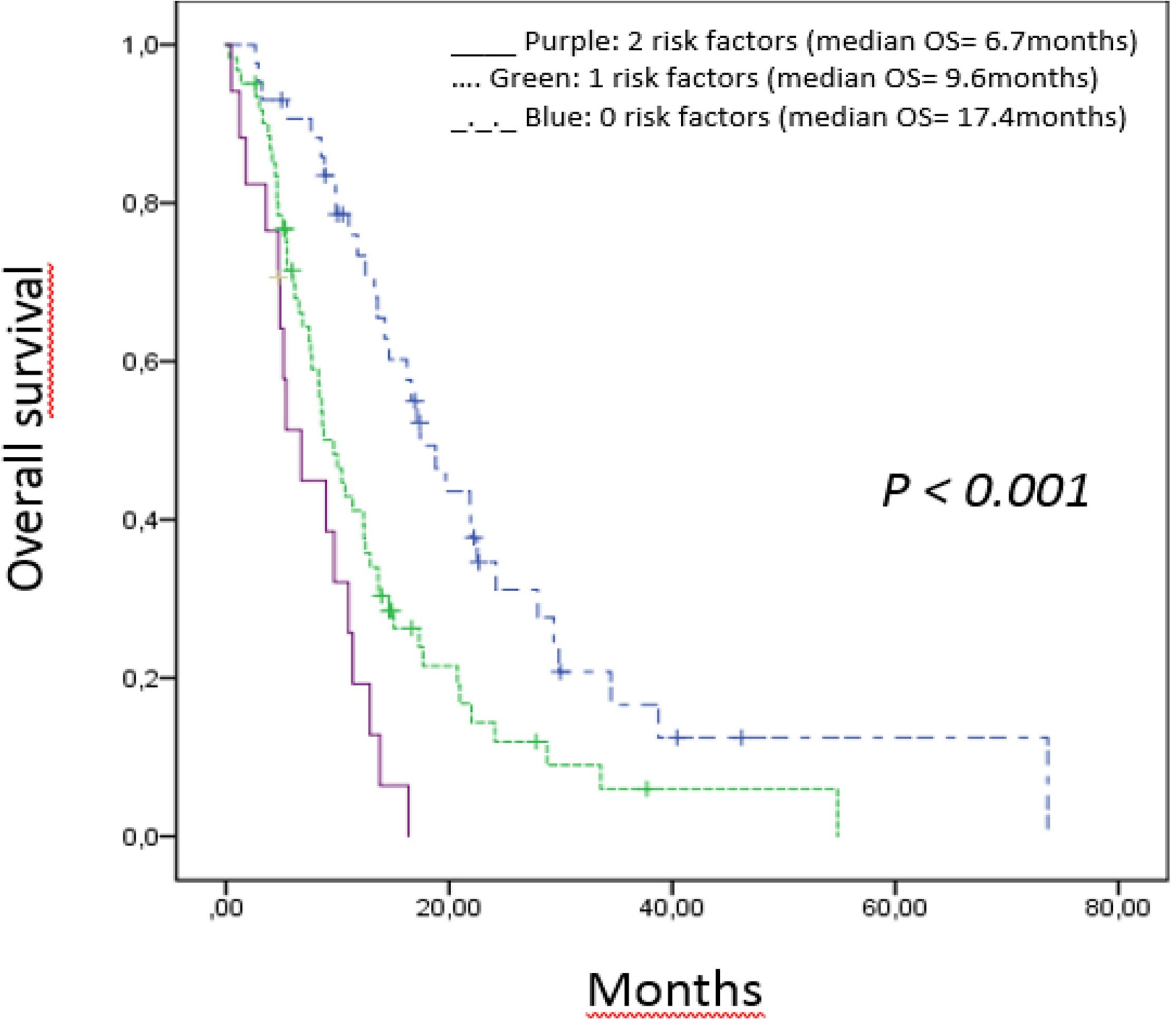
Overall survival score for all (n = 123) patients treated with FOLFIRINOX. The two risk factors CEA >4 and a BMI> 25 were found to be independent prognostic factors for overall survival. Purple line (2 risk factors): median OS 6.7 months. Green line (1 risk factors): median OS 9.6 months. Blue line (0 risk factors): median OS 17.4 months.

Of the 121 patients with complete data available for both variables, 43 patients had no risk factor, 51 patients had 1 risk factor and in 17 patients 2 risk factors could be identified. The median OS for these three categories were 17.4 months (95% CI), 9.6 (95% CI) months and 6.7 (95% CI) months, respectively (Fig 1). Survival differences between the groups were statistically significant (p<0.001). Table 2

**Table 2:** Prognostic factors for OS.

		Univariate			Multivariate		
Variable:		HR (95% CI)	P (1)	n	HR (95% CI)	P (1)	n
**CEA**	>4 mcg/l	2.1(1.44–3.34)	<0.001	122	1.9(1.17–3.23)	0.01	122
**CA19-9**	>400 U/ml	1.5 (1.01–2.27)	0.043	122	1.1(0.71–1.95)	0.519	122
**CRP**	>0,6 mg/dl	1.53(1.02–2.28)	0.036	122	1.1(0.66–1.84)	0.705	122
**BMI**	>25 kg/m^2^	2.06(1.27–3.32)	0.003	122	1.8(1.07–3.1)	0.026	122
**Age**	>66 vs. <66 years	1.13(0.76–1.68	0.52	123	n.a.		
**ECOG**	0/1 vs. 2/3	1.55(0.93–2.55)	0.08	123	n.a.		
**Gender**	Male vs. female	1 (0.67–1.48)	0.99	123	n.a.		
**Stage**	LPAC vs. MPAC	1.14 (0.75–1.73)	0.52	123	n.a.		
**Bilirubin**	>1.5mg/dL	1.24 (0.74–2.09)	0.4	123	n.a.		
**Leucocytes**	>10 G/L	1.3 (0.8–2.02)	0.24	123	n.a.		
**Neutrophiles**	>8G/L	1.3 (0.82–2.3)	0.2	122	n.a.		
**DM**	yes vs.no	1.13 (0.68–1.82)	0.63	122	n.a.		
**Stent**	yes vs.no	1.2 (0.52–2.67)	0.6	123	n.a.		
**NLR**	>6	1.06 (0.99–1.14)	0.068	123	n.a.		

Abbreviations: (1) cox regression analyses, Ci = confidence interval, n.a. = not available, LPAC = locally advanced pancreatic cancer, MPAC = metastatic pancreatic cancer, Vs. = versus, NLR = neutrophils to lymphocytes ratio, DM = diabetes mellitus

Cox regression analyses for the significant parameters to OS were also used for testing significance on PFS. None of the markers were statistically significant for PFS, however our 3 risk factor groups PFS curves separate suggesting a clinical relevance for treatment outcome as well.

### Adverse events

The major obstacle in applying polychemotheray regimens such as FOLFIRINOX is toxicity (3). Polyneuropathy (PNP) was the most common grade 3/4 adverse event and occurred in 10.5% of the patients (n = 13). PNP G3/4 was only reported with longer therapy duration, i.e. >5 chemotherapy applications. Diarrhea leading to hospital admission and subsequent dose reduction was reported in 7.3% of the patients (n = 9). Leukopenia and infectious complications were reported in 6.5% of the patients (n = 8) and 5.6% of the patients (n = 7), respectively. (See Table 3 for further details and comparison with the literature)

**Table 3:** Therapy related toxicities. Events comparison between our cohort and previously published by Conroy et al, NEJM, 2011.

Toxicity Grade 3/4 –no. (%)	Our cohort(N = 123)	Conroy et al, FOLFIRINOX cohort (N = 171)
**Leukopenia/Neutropenia**	7%	46%
**Diarrhea**	7%	13%
**Polyneuropathy**	11%	9%
**Infection**	6%	5%

The relatively modest rate of leukopenia as compared to the literature may have been due to wide usage of Granulocyte colony stimulating factor (G-CSF) support in 55.3% of the patients (n = 68). G-CSF from cycle one was administered to 55.3% of patients (n = 68) primarily for patients over the age of 66 and ECOG >1, respectively, which was associated with a statistically non-significant reduction in hospital readmissions during treatment with FOLFIRINOX of 50% as compared with the patients not receiving G-CSF.

Leukopenia, infections and diarrhea were seen especially during the first 4 doses of chemotherapy. Treatment had to be discontinued after the first chemotherapy application in 10 (12.3%) of the patients. Of those, 4.8% of the patients (n = 6) stopped treatment due to progressive disease, two patients died from infectious complication and one of cardiac arrest. Furthermore, one patient was switched to gemcitabine/ nab-paclitaxel due to toxicity and one patient was stopped due to his wish.

We attempted to define parameters for the development of toxicity in order to help identifying patients upfront who would be likely to suffer from toxicity and thus not having the benefit of the more aggressive chemotherapy regimen.

We defined time to first dose defining toxicity which was 4.6 months in median and possible risk factors for toxicity in uni- and multivariate analyses. Regarding risk factors for adverse events in uni- and multivariate analyses the same clinical factors as for our OS analyses, as listed in Table 3.

Thrombocytosis, defined as platelets elevated over 425 G/L by ROC analyses, and low BMI of <20 were statistically significant (p = 0.005) predictors for toxicity.

## Discussion

Polychemotherapy is the current standard of care for fit advanced pancreatic cancer patients as it significantly increases survival compared to monotherapy by gemcitabine [4,5]. Besides gemcitabine plus nab-paclitaxel, the FOLFIRINOX combination is one of the main options for treating pancreatic cancer patients. From reported data FOLFIRINIOX seems the more effective polychemotherapy regimen with regards to ORR, PFS, and OS [4]. However, toxicity is a major concern and has tempered the enthusiasm for its usage. Due to the high response and survival rates reported with FOLFIRINOX, we offered FOLFIRINOX to all eligible patients.

There are no oncologic practitioners in private practice in the Austrian health care system. Therefore, systemic treatment of patients with pancreatic cancer in Salzburg and surrounding regions is centralized to our department. In addition, nearly 100% of Austrians have a health assurance, chemotherapy is fully covered and socioeconomic biases may be lower than in other countries and our analysis is likely to be less biased regarding insurance status or other socioeconomic factors than one might assume in international randomized clinical trials. Thus our study is one of the largest single center retrospective analyses of a real world and relatively unselected patient collective with regards to comorbidities, age and performance status [12].

In this study, the median OS for patients treated in a palliative therapy setting with FOLFIRINOX was 11.8 months and the median PFS was 5.7 months. These data are consistent with the results reported in the literature, even though one third of our patients started their treatment course with dose reduction and following subsequent cycles. A reduced dose at the first and subsequent cycles was applied in 34% of the patients (n = 42 patients) resulting in a similar OS of 11.8 (95% CI) compared to 12.5 (95% CI) months in patients, who were treated with a full- dose regimen.

Given that there were no significant differences in PFS and OS between starting dose at full and at reduced dose the efficacy did not seem compromised by dose reductions. Recently there have been several phase 2 trials published, comparing a modified FOLFIRINOX regiment to full dose FOLFIRINOX in regard to decrease its side effects and increase its tolerability. A systematic review and meta- analyses done by Tonq H. et al showed a modified FOLFIRINOX could provide comparative survival benefits with fewer adverse events compared to the conventional dosage. Recently there have been several phase 2 trials published, comparing a modified FOLFIRINOX regiment to full dose FOLFIRINOX in regard to decrease its side effects and increase its tolerability. Our result is thus in line with other reports [13].

Side effects with regards to Grad 3 and 4 toxicity and hospitalization were reduced in our cohort compared to historical data. We observed PNP in 10.5% (13 patients), diarrhea 7.3% (9 patients), leukopenia 6.5% (8 patients), and infection 5.6% (7 patients). These values were significantly lower compared to the observation reported by Conroy et al. This may be explained by our observational cohort, since full toxicity reporting is difficult to implement in everyday practice. We are, however, confident that clinically relevant side effects have been adequately documented in our database. Our study suggests that implementation of dose modifications from the first cycle throughout therapy course and a wide usage of growth factor support for patients at risk for FUO is related to an overall improved tolerability. The use of 2^nd^ line chemotherapy was shown to be associated with an increase in OS in metaanalyses (Nagrial 2015) and was feasible after FOLFIRINOX in 71 (86%) of our patients (n = 71). This seems to be a higher percentage compared to the 47% of patients in the FOLFIRINOX arm of the PRODIGE4/ACCORD11study.

Despite advances in developing modern therapy compounds, the survival of patients with metastatic pancreatic cancer remains dismal and it is difficult to identify patients who derive optimal benefit from treatment [14]. There are no prospectively validated prognostic risk scores from large cohorts of pancreatic cancer patients treated with FOLFIRINOX recommended for use in clinical practice. Therefore, we aimed to develop an easily applicable risk score derived from commonly available laboratory and clinical parameters that may aid in risk stratification.

Our aim was to come up with an extended more detailed risk assessment of not only laboratory features but also integrating clinical ones, compared to the one already published in the literature [15,16,17,18,19]. Next to classical patient characteristics, tumor marker, and chronic inflammation markers we also added habitual disposition like body mass index in our detailed analysis of possible risk factors.

Overweight and obesity are not only well-known risk factor for the development of cardiovascular disease and diabetes but also for the development of several types of cancer including pancreatic cancer [20,21,22,23].

However, the influence of a higher BMI on outcome of patients with advanced pancreatic cancer is not known.

In the presence of gastric cancer a higher BMI has been reported of having a positive effect on OS, but not so for pancreatic cancer patients [24].

In the literature a prognostic effect of BMI remains unclear for patients under therapy for pancreatic cancer as published results are contradictory [25,26]. These contradictions may arise due to the fact that studies do not always analyze homogeneous patient populations in regard to inclusion of obese patients or obese and overweight ones.

Furthermore, the time points of BMI calculation vary throughout the literature from BMI measurement before curative surgery to BMI calculation at the time point of histologically established diagnosis through Whipple OP or biopsy. No data are available on the role of BMI for patients treated in a palliative therapy setting and in regard to applied drug regimen. Overweight is defined as BMI of 25.0 to 29.9kg/m^2^. BMI above 29.9kg/m^2^ is obesity [10]. When we used standardized WHO definitions of overweight and obese our analyses detected only seven obese patients. Therefore we concentrated on the overweight study population in more detail. Distribution between BMI categories widely differs between central Europe and the USA. A recent metaanalysis of PC done by Shi et al [25] could find a prognostic worsening of OS only for obese patients, however not for overweight patients in the US population.

We used commonly available clinical and laboratory patient characteristics for prediction of either benefit in regard to tumor control by the FOLFIRINOX regimen or the experience of severe toxicity. In univariate analyses elevated CEA & CRP, CA19.9 > 400, and a BMI>25, were significantly associated with OS. The upper limit of normal for Ca 19–9 is 35 U/ml in our laboratory, however we could not show a statistically significance for OS with CA 19–9 levels above 35U/ml (p = 0.320). Therefore we went for clustering Ca 19–9 levels and took the cut off for CA 19–9 at 400U/ml. This cut off is arbitrary, but was also used by Safi at al.

A multivariate regression analyses was conducted including these parameters and BMI>25 and CEA baseline were confirmed an independent prognostic value. Factors with independent prognostic power in the cox regression analyses were used to build up a score separating patient groups with different survival. A prognostic score was generated for all patients by attributing one point for each of the values. The median OS was 17.4, 9.6 and 6.7 months respectively for patients with 0, 1 or 2, risk factors respectively (p<0,001).

Furthermore we identified thrombocytosis and a low BMI at start of therapy as risk factors for toxicity.

Thrombocytosis as a surrogate for paraneoplastic phenomena and inflammation, has been shown to have a negative influence on overall survival in various cancer patients due to their role in hematogenous dissemination of cancer cells. One molecular mechanism might be that activated platelets not only promote primary cancer growth by releasing a large number of growth factors (VEGF, EDGF) but also help in forming cancer aggregates. These aggregates play a role in immune response evasion and metastatic implantation in other organs by modifying blood stream which is harmful to cancer cells. Thrombocytosis as part of chronic inflammation status may interfere with drug metabolism and therefore causing more toxicity, however we do not know exact pathophysiological mechanisms.

A low BMI reflects, weight loss, sarcopenia and tumor cachexia in cancer patients.

Sarcopenia has been used as an independent predictor of clinical outcomes in gastrointestinal cancers as a proxy for frailty and nutritional status.

Sarcopenia in general is a well known surrogate marker for worse postoperative outcome after pancreatic surgery. A plausible explanation for the excess of toxicity of sarcopenic patients is the usual practice of dosing chemotherapy as function of each patient’s height and weight. However, this is not taking the muscles and fat mass distribution into consideration in an appropriate way and without considering that fat mass accounts for a large and unpredictable part of body weight representing the volume of distribution of many cytotoxic chemotherapeutic agents. Sarcopenia helps physicians to address upfront-expected complications and side effects of pancreatic cancer patients like e.g. pain, fatigue, and nausea.

## Conclusion

In summary, we were able to identify two novel independent clinical and serum factors (CEA and BMI>25) that influence survival of pancreatic cancer patients treated with FOLFIRINOX. By combining these two factors to create a score that predicts OS, it is possible to distinguish a group with favorable prognoses and one with a worse outcome. It is also more evident according to our data that high BMI negatively affects OS in pancreatic cancer. Further, our proposed score predicts toxicity in regard to Grade 3–4 in combination with thrombocytosis and reduced body weight.

## Supporting information

S1 Table List of applied chemotherapy agents. List of applied chemotherapy agents in second line (FOLFIRINOX, Gemcitabine and Nab-paclitaxel, Nanoliposomal Irinotecan, Nab-paclitaxel, Gemcitabine- Erlotinib, Gemcitabine- Oxaliplatin, Docetaxel, Doxorubicin).
(DOCX)

S1 Data (XLSX)
