# Supplementary material for: Novel models for prediction of benefit and toxicity with FOLFIRINOX treatment of pancreatic cancer using clinically available parameters
Source: PLoS One. 2018 Nov 9;13(11):e0206688. doi: 10.1371/journal.pone.0206688 (PMC6226156; doi:10.1371/journal.pone.0206688)
Supplement: S1 Table — List of applied chemotherapy agents in second line (FOLFIRINOX, Gemcitabine and Nab-paclitaxel, Nanoliposomal Irinotecan, Nab-paclitaxel, Gemcitabine- Erlotinib, Gemcitabine- Oxaliplatin, Docetaxel, Doxorubicin). (DOCX) [file pone.0206688.s001.docx]

# Supporting information

*S1 Table;* List of applied chemotherapy agents

|  |
| --- |
| FOLFIRINOX |
| Gemcitabine and Nab-paclitaxel |
| Nanoliposomal Irinotecan |
| Nab-paclitaxel |
| Gemcitabine- Erlotinib |
| Gemcitabine- Oxaliplatin |
| Docetaxel |
| Doxorubicin |
